# Mammographic density does not correlate with Ki-67 expression or cytomorphology in benign breast cells obtained by random periareolar fine needle aspiration from women at high risk for breast cancer

**DOI:** 10.1186/bcr1683

**Published:** 2007-05-30

**Authors:** Qamar J Khan, Bruce F Kimler, Anne P O'Dea, Carola M Zalles, Priyanka Sharma, Carol J Fabian

**Affiliations:** 1 University of Kansas Medical Center, 3901 Rainbow Boulevard, Kansas City, Kansas 66160, USA

## Abstract

**Background:**

Ki-67 expression is a possible risk biomarker and is currently being used as a response biomarker in chemoprevention trials. Mammographic breast density is a risk biomarker and is also being used as a response biomarker. We previously showed that Ki-67 expression is higher in specimens of benign breast cells exhibiting cytologic atypia that are obtained by random periareolar fine needle aspiration (RPFNA). It is not known whether there is a correlation between mammographic density and Ki-67 expression in benign breast ductal cells obtained by RPFNA.

**Methods:**

Included in the study were 344 women at high risk for developing breast cancer (based on personal or family history), seen at The University of Kansas Medical Center high-risk breast clinic, who underwent RPFNA with cytomorphology and Ki-67 assessment plus a mammogram. Mammographic breast density was assessed using the Cumulus program. Categorical variables were analyzed by χ^2 ^test, and continuous variables were analyzed by nonparametric test and linear regression.

**Results:**

Forty-seven per cent of women were premenopausal and 53% were postmenopausal. The median age was 48 years, median 5-year Gail Risk was 2.2%, and median Ki-67 was 1.9%. The median mammographic breast density was 37%. Ki-67 expression increased with cytologic abnormality (atypia versus no atypia; *P *≤ 0.001) and younger age (≤50 years versus >50 years; *P *≤ 0.001). Mammographic density was higher in premenopausal women (*P *≤ 0.001), those with lower body mass index (*P *< 0.001), and those with lower 5-year Gail risk (*P *= 0.001). Mammographic density exhibited no correlation with Ki-67 expression or cytomorphology.

**Conclusion:**

Given the lack of correlation of mammographic breast density with either cytomorphology or Ki-67 expression in RPFNA specimens, mammographic density and Ki-67 expression should be considered as potentially complementary response biomarkers in breast cancer chemoprevention trials.

## Introduction

Established risk factors for the development of breast cancer include components incorporated into the Gail model, breast mammographic density, and cellular atypia. Mammographic density is an important biomarker of risk for the development of breast cancer and, because it is modifiable, it is a potential response biomarker as well. Cytologic atypia is an established risk factor for the development of breast cancer. A prospective study conducted in high-risk women employed random periareolar fine needle aspiration (RPFNA) to sample breast tissue [1]. It revealed that women with RPFNA atypia had a fivefold increased risk for subsequent clinical development of ductal carcinoma *in situ *(DCIS) or invasive cancer as compared with those without atypia, and RPFNA atypia stratified risk based on the Gail model [1]. Both mammographic density and cellular atypia are risk biomarkers that can stratify estimates based on the Gail model but they have limitations, particularly when they are used as surrogate markers of response, which include interpretive variance (both biomarkers), lack of categorical change (cellular atypia), and lack of change with some effective interventions compared with placebo in postmenopausal women (mammographic density) [2,3].

Increased proliferation is a fundamental process in carcinogenesis. Shabaan and coworkers [4], in a cross-sectional study, observed that women with increased Ki-67 in foci of hyperplasia were at increased risk for breast cancer. Reduction in proliferation has been shown to correlate with response to antihormonal agents in cancer treatment trials [5]. Ki-67 expression is currently being used in phase II breast cancer chemoprevention trials. The rationale behind the use of Ki-67 as a response biomarker in phase II proof-of-principle trials would be stronger if correlation could be established with development of cancer or with other biomarkers associated with a substantial increase in risk (atypical morphology and high mammographic density, for instance). On the other hand, if no strong correlation could be established, then these two biomarkers may be regarded as independent and potentially complementary risk or response biomarkers. We have previously shown that Ki-67 expression in benign epithelial cells is positively correlated with epithelial cell number and cytomorphologic abnormality in women at increased risk for breast cancer [6]. In this analysis, we examine the correlation between Ki-67 and mammographic density.

## Materials and methods

### Study cohort

The study cohort consisted of high-risk women undergoing baseline eligibility assessment for one of several prevention or surveillance trials at the University of Kansas Medical Center Breast Cancer Prevention Center. Women eligible for RPFNA were those with one of the following risk factors: one first-degree relative or more with breast cancer diagnosed at less than 60 years of age; multiple second-degree relatives with breast cancer; known carrier of a mutation in *BRCA1 *or *BRCA2*; 5-year Gail risk of 1.7% or those whose relative risk for developing breast cancer was at least three times that in the general population; prior breast biopsy that had exhibited atypical hyperplasia or lobular carcinoma *in situ*; and having undergone treatment for a prior contralateral invasive breast cancer or DCIS. All women had a normal mammogram at the time of aspiration and had undergone no change in hormone replacement therapy (HRT) or ingested any selective estrogen receptor modulator or aromatase inhibitor for a period of 6 months before RPFNA. They were required also to have been at least 1 year from pregnancy, lactation, or any prior chemotherapy. This study was performed after approval by the University of Kansas Medical Center Human Subjects Committee. The participants signed an informed consent form before each breast aspiration.

### Ki-67 and cytomorphology by random periareolar fine needle aspiration

RPFNA was performed to obtain breast epithelial cells under local anesthesia from two sites (upper outer and upper inner quadrant) and cells were pooled from both breasts [1]. Women with a prior history of DCIS or invasive breast cancer had RPFNA done only on the uninvolved breast. For premenopausal women, all RPFNAs were done on days 1 to 12 (follicular portion) of the menstrual cycle.

Material from all breast aspiration sites for each woman was pooled in a 15 ml conical tube containing 9 ml CytoLyt (Cytyc, Boxborough, MA, USA) and 1 ml of 10% neutral buffered formalin. Conical tubes were placed on a Verimix Rocker (Barnstead International, Dubuque, IA, USA) at low speed. Cells were then washed with CytoLyt, processed to a pellet, placed in PreservCyt (Cytyc) for 48 hours, and then processed to at least three slides using a standard Thin Prep 2000 (Cytyc) nongynecologic protocol. At least two slides were Papanicolaou stained, with one used for morphology and one for Ki-67 staining. Slides for both cytomorphology and Ki-67 were Papanicolaou stained under RNase-free conditions with hematoxylin, OG-6 and EA-65 (all from Richard Allen Scientific, Kalamazoo, MI, USA), and were prepared on the Thin Prep Processor. Cytomorphology was assessed by a single cytopathologist (CMZ), who assigned a categorical assessment of nonproliferative, hyperplasia, borderline hyperplasia with atypia, or hyperplasia with atypia [7,8], and a Masood semiquantitative index score [9]. Cytologic assessments were made without knowledge of the results of the Ki-67 assessment. Only slides in which more than 500 epithelial cells were visible by Papanicolaou staining were further processed for Ki-67. After de-staining, antigen retrieval was performed with a 10 nmol/l citrate buffer (pH 6) in a Biocare (Walnut Creek, CA, USA) decloaking chamber (DC 2002) for 2 min at 120°C. Slides were stained with a MIB-1 monoclonal antibody (M7240 Dako Cytomation; Dako, Carpenteria, CA, USA) at a 1:20 dilution using a Dako autostainer [6]. Hyperplastic clusters were preferentially assessed, and the number of cells with unequivocal nuclear staining out of a total of 500 cells was assessed manually by two technicians and a consensus score recorded.

### Breast mammographic density assessment

Mammography was performed within 6 months of the RPFNA procedure. Cranio-caudal views of mammograms were digitized using a Lumisys Lumiscan 85 (Lumisys Inc., Sunnyvale, CA, USA). Left mammogram was used for assessment except in women with prior cancer, in which the mammogram of the unaffected breast was used. All mammograms were assessed by a single operator (QJK) using the Cumulus computer-assisted program [10]. The parameters recorded were the total breast area and the area of breast considered to be at increased density (both in pixels and cm^2^). The percentage of the breast in which density was increased was then calculated. One mammogram from every batch was a duplicate. Because there were 12 batches, 12 mammograms were read twice to determine reproducibility. The *R*^2 ^for reproducibility was 0.88.

### Statistical analysis

Frequencies of categorical variables were assessed using χ^2 ^analysis. Continuous variables were assessed using the Mann-Whitney or Kruskal-Wallis nonparametric test. Multivariate analyses were conducted using stepwise linear regression.

## Results

Included in this analysis were all women at high risk for breast cancer who had undergone RPFNA for risk assessment or eligibility assessment for one of several clinical chemoprevention trials between March 2003 and May 2006. This provided 344 evaluable individuals for whom sufficient ductal cells (>500) were present in the slide designated for Ki-67 and who had a mammogram for density measurement. Median age was 48 years (range 20 to 78 years). Median height was 1.65 m (1.5 to 1.8 m) and median weight was 68 kg (43 to 119 kg). One hundred and sixty-two (47%) women were premenopausal and 182 (53%) were postmenopausal. Among postmenopausal women, 114 (63%) women were on some form of HRT, including 78 on estrogen alone, nine on estrogen plus testosterone, 27 on estrogen plus progestins (eight with testosterone as well).

### Ki-67 expression

Median level of Ki-67 expression was 1.9% (range 0% to 33%). There was excellent agreement and low interobserver variance between the two readers for the 344 specimens (*R*^2 ^= 0.99). Median Ki-67 was 3.3% among specimens from premenopausal women as compared with 1.2% in specimens from postmenopausal women (*P *< 0.001; Table 1). There was no difference in Ki-67 expression between specimens from postmenopausal women receiving HRT and specimens from those who were not on HRT, but only 21% of women on HRT were on estrogen plus a progestin. Ki-67 expression was greater in specimens exhibiting cytomorphologic atypia (*P *= 0.01) and greater cellularity (*P *= 0.001; Table 2).

**Table 1:** Correlation of Ki-67 and mammographic density with demographics and 5-year Gail risk

Variable	Number (%)	Median (interquartile range)			
		
		Ki-67 (%)	*P*	Mammographic density (%)	*P*
Total	344	1.9 (0.4–4.6)		37 (21–54)	
Age (years)					
<48	170	3.1 (1.2–6.0)	<0.001	40 (23–57)	0.035
>48	174	1.0 (0.2–3.4)		35 (19–52)	
Menopause status					
Pre/peri	162 (47%)	3.3 (1.0–5.8)	<0.001	45 (30–64)	<0.001
Post	182 (53%)	1.2 (0.2–3.6)		32 (16–47)	
Post and HRT	114 (33%)	1.2 (0.2–4.0)	0.63	35 (20–49)	0.049
Post but no HRT	68 (20%)	1.0 (0.2–3.2)		24 (11–41)	
BMI (kg/m^2^)					
<25	166	2.0 (0.6–4.6)	0.76	49 (34–66)	<0.001
>25	178	1.8 (0.4–4.4)		29 (11–42)	
5-year Gail risk (%)					
<2.2	167	2.6 (1.0–6.0)	<0.001	43 (28–58)	0.001
>2.2	176	1.2 (0.2–3.6)		33 (17–51)	
Live birth					
Yes	63	2.2(0.2–5.4)	0.65	45 (32–76)	0.001
No	281	1.8 (0.6–4.4)		36 (30–51)	
Prior breast cancer					
No	305	2.0 (0.6–4.8)	0.027	38 (22–55)	0.12
Yes	39	1.0 (0.2–3.0)		33 (16–50)	

The median (range) age was 48 years (20 to 78 years), the median (range) body mass index (BMI) was 25 kg/m^2 ^(17 to 44 kg/m^2^), and the median (range) 5-year Gail risk was 2.2% (0% to 16%). BMI, body mass index; HRT, hormone replacement treatment.

**Table 2:** Correlation of Ki-67 and mammographic density with cytomorphology

Variable	Number (%)	Ki-67 (median [interquartile range])	*P*	Mammographic density (median [interquartile range])	*P*
Cytomorphology					
No atypia	226 (65%)	1.2 (0.2–3.6)	<0.001	37 (20–54)	0.81
Hyperplasia with atypia	118 (35%)	3.6 (1.4–5.8)		37 (22–55)	
Cytomorphology (Masood score)					
11	11 (3%)	0.6 (0–1.8)	0.001^a^, 0.001^b^	37 (21–58)	
12	54 (16%)				0.92^c^
13	64 (19%)	1.6 (0.4–4.2)		37 (20–53)	
14	106 (31%)				
15	95 (28%)	3.4 (1.4–5.4)		38 (22–55)	
≥16	14 (4%)				

^a^Masood score 11/12 versus 13/14. ^b^Masood score 13/14 versus 15/16. ^c^Masood score 11/12 versus 15/16.

### Mammographic density

**Figure 1:**
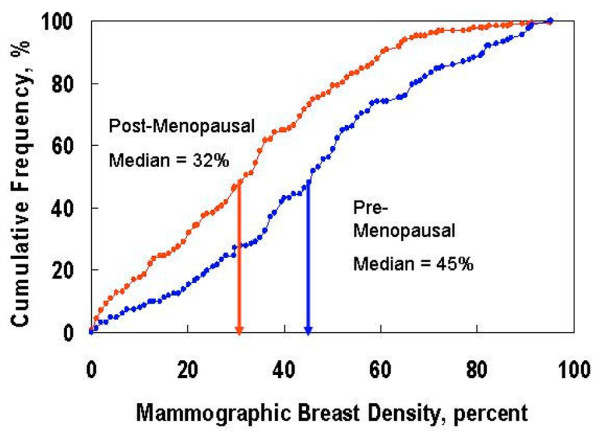
Cumulative frequency of mammographic density for premenopausal and postmenopausal women at high risk for breast cancer.

The median mammographic density for the entire cohort was 37% (range 0% to 95%). Mammographic density was higher in premenopausal women than in postmenopausal women (Table 1). Median mammographic density was 45% in premenopausal women as compared with 32% in postmenopausal women (*P *< 0.0001; Figure 1). Among postmenopausal women, median mammographic densities were as follows: 24% in 68 women receiving no HRT; 30% in 78 women taking estrogen alone; 59% in women taking progestins alone (only four women); 36% in 16 women taking a combination of an estrogen and a progestin; and 47% in eight women taking an estrogen, a progestin and testosterone. The difference between estrogen alone (32% [85 women]) and estrogen plus a progestin (40% [24 women]) was of borderline significance using the nonparametric test (*P *= 0.076) but it was not significant in multivariate analysis. Mammographic density was higher in younger women (declining from a median of 61% for women in their 20s to 29% for women older than 60 years; *P *= 0.012), in women with a lower 5-year Gail risk (43% for women with risk <2.2% versus 34% for women with risk ≥2.2%, *P *= 0.001) and women with lower body mass index (BMI; 49% for women with BMI <25 kg/m^2 ^versus 29% for women with BMI ≥25 kg/m^2^; *P *< 0.001). On multivariate analysis, mammographic density was significantly higher in women with a lower BMI (*P *< 0.0001), premenopausal women (*P *= 0.002) and women with lower Gail risk (*P *= 0.004).

### Correlation of mammographic density with Ki-67 and cytomorphology

**Figure 2:**
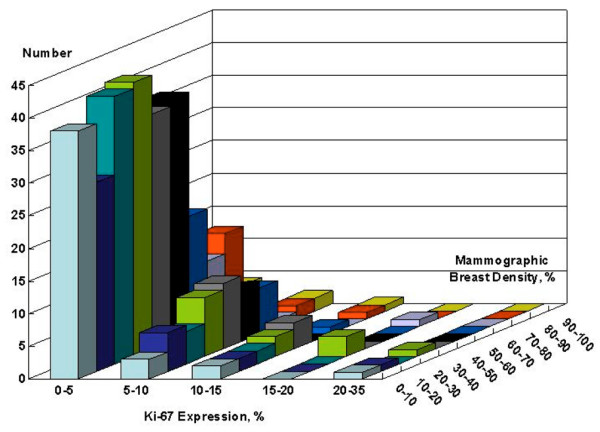
Lack of correlation between Ki-67 expression and mammographic breast density.

No correlation was seen between percentage mammographic density and Ki-67 by multivariate analysis using stepwise linear regression (*P *= 0.82; Figure 2). Other variables included in the analysis were age, menopausal status, 5-year Gail risk, HRT status, BMI, height and weight. Similarly, no association was observed between mammographic density and cytomorphology by traditional methods (atypia versus no atypia), Masood semiquantitative method and National Cancer Institute consensus panel criteria. Using the traditional method, median mammographic density was 36.8% in women with epithelial hyperplasia and 37.5% in women with hyperplasia and atypia (*P *= 0.81; Table 2). With the Masood semiquantitative index, median mammographic densities were 30%, 37%, 40%, 35%, 38% and 44% for Masood scores of 11, 12, 13, 14, 15 and 16, respectively (*P *= 0.61). Sixty-five women had a Masood score of 11 and 12 (lowest category) and 109 had scores of 15 and 16 (highest category). There was no difference in mammographic density between the lowest and the highest Masood category. By multivariate analysis, Ki-67 was higher in specimens with atypia (*P *< 0.001) and younger women (*P *< 0.001). However, age is highly associated with menopausal status and Gail risk. If age was omitted from the model, then Gail risk (*P *= 0.009) and menopausal status (*P *= 0.029) exhibited significant association with Ki-67 in addition to atypia. Table 3 shows the results of linear regression analysis for Ki-67 and for mammographic density.

**Table 3:** Results of linear regression analysis

Dependent variable	Independent variable	*P*
Ki-67	Cell number on slide	0.001
	Age at RPFNA (inverse)	0.001^a^
Breast density (%)	BMI	<0.001
	No live birth	<0.001
	Premenopausal	0.004
	5-year Gail risk	0.02

^a^If age is omitted, then Gail risk (strong age component) enters as an independent variable (*P *= 0.012), followed by atypia (*P *= 0.012) and premenopausal status (*P *= 0.036). BMI, body mass index; RPFNA, random periareolar fine needle aspiration.

## Discussion

Breast mammographic density is reflective of the amount of epithelium, stroma and breast fluid relative to fat (which is radiolucent). The volume of stroma and collagen in most women influences density to a greater extent than the amount of breast epithelium [11,12]. Mammographic density is positively associated with several other risk factors and biomarkers, including breast intraepithelial neoplasia [13], serum insulin-like growth factor-I and growth hormones in premenopausal women [14], serum prolactin and combined estrogen plus progestin HRT in postmenopausal women [15,16], and family history of breast cancer [17].

Boyd and coworkers [10], in a case control study using computer-assisted measurements, found that statistically significant increases in breast cancer risk were associated with increasing mammographic density. The increment in relative risk for breast cancer for each percentage increase in density was 2% (*P *< 0.0001) and the relative risk for greater than 75% density relative to no density was 4.04 (95% confidence interval 2.12 to 7.69). Breast density is favorably modulated by some but not all drugs/interventions that are effective in the prevention and adjuvant treatment of breast cancer. Tamoxifen was associated with a 14% reduction in absolute breast density over 54 months, as compared with an 8% reduction for placebo-treated women in the IBIS-1 (International Breast Cancer Intervention Study-1) trial [18], in a cohort of women with greater than 10% density. Changes in mammographic density favoring tamoxifen were significant only in premenopausal women and those under the age of 55 years. Similarly, two years of a low-fat diet was demonstrated to reduce the area of breast density for premenopausal but not postmenopausal women [19], despite the observation in the WINS (Women's Interventional Nutritional Study) study [20] that such an intervention significantly reduced the risk for recurrence, including contralateral breast cancer, in postmenopausal women [20]. Although breast density is clearly a risk factor for both premenopausal and postmenopausal women, its accuracy in predicting response to a preventive intervention is less clear, particularly for postmenopausal women. It seems plausible, based on the evidence, that interventions associated with reduced density are likely to be effective preventive agents.

Because prospective prevention intervention studies with cancer as an end-point are expensive and lengthy, surrogate response biomarkers are often used in phase II chemoprevention trials, in which favorable modulation of biomarker by an agent is taken as support for that agent's ability to reduce the incidence of cancer. A response biomarker ideally should also be a risk biomarker in addition to being modifiable. Mammographic density is an established risk factor for breast cancer, as noted above, and is modifiable. Ki-67 expression in benign breast cells obtained from RPFNA is also a reversible, a potential risk and a possible surrogate response biomarker. In a cohort of 147 high risk women, we previously showed that cytomorphologic atypia in benign breast cells obtained by RPFNA is associated with increased Ki-67 expression [6]. Median Ki-67 expression was 2.8% in women with RPFNA atypia as compared with 1.1% in women without atypia. In the present study, which now includes 344 women, this correlation between Ki-67 and cytomorphologic atypia persists and corroborates our previously reported data. Whereas proliferation appears to be linked to cytologic atypia, it is not clear whether there is a link between mammographic density and proliferation or cytomorphology. Study of such a correlation is important because both mammographic density and Ki-67 are currently being used in breast cancer prevention trials as surrogate response biomarkers [21,22].

We found no correlation between mammographic density and Ki-67 or mammographic density and cytomorphologic atypia in benign breast cells in a cohort of high-risk women for whom sufficient cells from RPFNA were available for both cytomorphology and Ki-67 testing. No study has previously been undertaken to identify such a correlation. Many epidemiologic studies have evaluated a relationship between mammographic density and benign breast histology, with some studies showing an association between histology and mammographic density, whereas others have shown no such association. In a cohort of women taking part in the Canadian NBSS (National Breast Screening Study) study [23], proliferative breast disease was found to be more frequent in women with greater breast density. A similar association was noted in a study reported by Bland and coworkers [24]. Two other studies [25,26], on the other hand, identified no correlation between histology and mammographic density. In a nested case-control study within the prospective Breast Cancer Detection Demonstration Project, percentage mammographic density and benign breast disease histology were found to be distinct breast cancer risk factors. The risk associated with benign breast disease was not explained by the effects of percentage breast density, and the risk associated with percentage breast density was not explained by benign breast histology, suggesting a lack of correlation between the two risk factors [27]. Several cross-sectional studies have described an association between histology and mammographic density, with different results [25,28-31]. Fisher and coworkers [28] compared histology and mammographic appearance of breast in women with cancer and women with fibrocystic disease, and found no association between epithelial change and mammographic density. They found that mammographic densities were associated with fibrosis in breast parenchyma. A similar lack of association was described by another study [25]. In contrast to these studies finding no association, Bright and coworkers [30] reported associations between mammographic density and epithelial hyperplasia when xerographic and histologic findings in women with benign breast disease were compared. Similarly, Urbanski and colleagues [31] described an association between atypia and extensive mammographic density.

Association between proliferation in benign breast and mammographic density is less well studied. In a recently reported study, Ki-67 (MIB-1) expression was assessed in areas of low, medium, and high mammographic density in benign breast tissue obtained from reduction mammoplasties. Contrary to what might be expected, Ki-67 expression in epithelial cells was less in the areas of medium and high density as compared with the areas of low density [32]. In another prospective study of association between mammographic density and benign histology [33], mammographically dense and nondense (fatty) tissues contained similar frequencies of hyperplasia with atypia and proliferative activity, as determined by S-phase percentage. These latter observations suggest a lack of strong correlation between mammographic density and proliferative activity within the breast. Our findings with random tissue sampling are consistent with these findings.

Our cohort of 344 women includes 114 women who were on HRT, which could be a potential confounding factor. However, only 16 women were taking a combination of an estrogen and a progestin. Combined estrogen plus progestin HRT, and not estrogen alone, is associated with increases mammographic density in postmenopausal women [15,16]. We therefore do not believe that HRT status had any significant impact on our results, namely a lack of correlation between mammographic density and Ki-67 expression. Our cohort also included 39 women with a history of prior breast cancer. Cancer treatment such as endocrine therapy or premature menopause from chemotherapy could potentially have a confounding effect on our results. However, only 11 women in our cohort had invasive cancer, eight women received chemotherapy, and four women took tamoxifen. Given the small number of women receiving interventions that could have confounding effect on mammographic density, we do not believe that inclusion of these women in the cohort influenced our findings. Furthermore, we ran an analysis excluding these women with prior cancer and there was no difference in the results.

## Conclusion

Our findings indicate that mammographic density, cytomorphology, and Ki-67 expression are independent variables, and may be complementary when used as risk predictors or response biomarkers in breast cancer chemoprevention trials. Furthermore, our results continue to show that Ki-67 expression is associated with the risk biomarker cytomorphology in high-risk women, and thus they provide evidence that Ki-67 may be used as a response biomarker in proof-of-principle phase II trials.

## Abbreviations

BMI = body mass index; DCIS = ductal carcinoma *in situ*; HRT = hormone replacement therapy; RPFNA = random periareolar fine needle aspiration.

## Competing interests

The authors declare that they have no competing interests.

## Authors' contributions

QJK contributed to the study design, read all the mammograms and drafted the manuscript. BFK performed the statistical analysis and contributed to the study design. AOD organized and collected the data for analysis. CMZ made all the cytology assessments. PS performed RPFNA to obtain cytology specimens. CJF performed RPFNA, contributed to the study design, and served as a mentor for the entire project.

## References

[B1] Fabian CJ, Kimler BF, Zalles CM, Klemp JR, Kamel S, Zeiger S, Mayo MS (2000). Short-term breast cancer prediction by random periareolar fine-needle aspiration cytology and the Gail risk model. J Natl Cancer Inst.

[B2] Boyd NF, Greenberg C, Lockwood G, Little L, Martin L, Byng J, Yaffe M, Tritchler D (1997). Effects at two years of a low-fat, high-carbohydrate diet on radiologic features of the breast: results from a randomized trial. Canadian Diet and Breast Cancer Prevention Study Group. J Natl Cancer Inst.

[B3] Cuzick J, Warwick J, Pinney E, Warren RM, Duffy SW (2004). Tamoxifen and breast density in women at increased risk of breast cancer. J Natl Cancer Inst.

[B4] Shaaban AM, Sloane JP, West CR, Foster CS (2002). Breast cancer risk in usual ductal hyperplasia is defined by estrogen receptor-alpha and Ki-67 expression. Am J Pathol.

[B5] Dowsett M, Ebbs SR, Dixon JM, Skene A, Griffith C, Boeddinghaus I, Salter J, Detre S, Hills M, Ashley S (2005). Biomarker changes during neoadjuvant anastrozole, tamoxifen, or the combination: influence of hormonal status and HER-2 in breast cancer: a study from the IMPACT trialists. J Clin Oncol.

[B6] Khan QJ, Kimler BF, Clark J, Metheny T, Zalles CM, Fabian CJ (2005). Ki-67 Expression in benign breast ductal cells obtained by random periareolar fine needle aspiration. Cancer Epidemiol Biomarkers Prev.

[B7] Zalles C, Kimler BF, Kamel S, McKittrick R, Fabian CJ (1995). Cytology patterns in random aspirates from women at high and low risk for breast cancer. Breast J.

[B8] Zalles CM, Kimler BF, Simonsen M, Clark JL, Metheny T, Fabian CJ (2006). Comparison of cytomorphology in specimens obtained by random periareolar fine needle aspiration and ductal lavage from women at high risk for development of breast cancer. Breast Cancer Res Treat.

[B9] Masood S, Frykberg ER, McLellan GL, Scalapino MC, Mitchum DG, Bullard JB (1990). Prospective evaluation of radiologically directed fine-needle aspiration biopsy of nonpalpable breast lesions. Cancer.

[B10] Boyd NF, Byng JW, Jong RA, Fishell EK, Little LE, Miller AB, Lockwood GA, Tritchler DL, Yaffe MJ (1995). Quantitative classification of mammographic densities and breast cancer risk: results from the Canadian National Breast Screening Study. J Natl Cancer Inst.

[B11] Warren R, Lakhani SR (2003). Can the stroma provide the clue to the cellular basis for mammographic density?. Breast Cancer Res.

[B12] Haars G, van Noord PA, van Gils CH, Peeters PH, Grobbee DE (2004). Heritable aspects of dysplastic breast glandular tissue (DY). Breast Cancer Res Treat.

[B13] Boyd NF, Jensen HM, Cooke G, Han HL (1992). Relationship between mammographic and histological risk factors for breast cancer. J Natl Cancer Inst.

[B14] Byrne C, Colditz GA, Willet WC, Speizer FE, Pollack M, Hankinson SE (2000). Plasma insulin-like growth factor (IGF) I, IGF-binding protein 3, and mammographic density. Cancer Res.

[B15] Greendale GA, Reboussin BA, Sie A, Singh HR, Olson LK, Gatewood O, Bassett LW, Wasilauskas C, Bush T, Barrett-Connor E (1999). Effects of estrogen and estrogen-progestin on mammographic parenchymal density. Postmenopausal Estrogen/Progestin Interventions (PEPI) Investigators. Ann Intern Med.

[B16] Boyd NF, Stone J, Martin LJ, Jong R, Fishell E, Yaffe M, Hammond G, Minkin S (2002). The association of breast mitogens with mammographic densities. Br J Cancer.

[B17] Boyd NF, Dite GS, Stone J, Gunasekara A, English DR, McCredie MR, Giles GG, Tritchler D, Chiarelli A, Yaffe MJ (2002). Heritability of mammographic density, a risk factor for breast cancer. N Engl J Med.

[B18] Cuzick J, Warwick J, Pinney E, Warren RM, Duffy SW (2004). Tamoxifen and breast density in women at increased risk of breast cancer. J Natl Cancer Inst.

[B19] Boyd NF, Greenberg C, Lockwood G, Little L, Martin L, Byng J, Yaffe M, Tritchler D (1997). Effects at two years of a low-fat, high-carbohydrate diet on radiologic features of the breast: results from a randomized trial. Canadian Diet and Breast Cancer Prevention Study Group. J Natl Cancer Inst.

[B20] Chlebowski RT, Blackburn GL, Elashoff RE, Thomson C, Goodman MT, Shapiro A, Giuliano AE, Karanja N, Hoy MK, Nixon DW (2005). Dietary fat reduction in postmenopausal women with primary breast cancer: Phase III Women's Intervention Nutrition Study (WINS) [abstract 10]. J Clin Oncol.

[B21] Mincey BA, Perez EA (2004). Advances in screening diagnosis and treatment of breast cancer. Mayo Clin Proc.

[B22] Fabian CJ, Kimler BF, Simonsen M, Metheny T, Zalles C, Hall M (2004). Reduction in breast epithelial cell proliferation after six months of letrozole in high risk women on hormone replacement therapy with random periareolar fine needle aspiration evidence of atypia [abstract 5]. Breast Cancer Res Treat.

[B23] Boyd NF, Jensen HM, Cooke G, Han HL, Lockwood GA, Miller AB (2000). Mammographic densities and the prevalence and incidence of histologic types of benign breast disease. Eur J Cancer Prev.

[B24] Bland KI, Kuhns JG, Buchanan JB, Dwyer PA, Heuser LF, O'Connor CA, Gray LA, Polk HC (1982). A clinicopathologic correlation of mammographic parenchymal patterns and associated risk factors for human mammary carcinoma. Ann Surg.

[B25] Moskowitz M, Gartside P, McLaughlin C (1980). Mammographic patterns as markers for high risk benign breast disease and incidence cancers. Radiology.

[B26] Arthur JE, Ellis IO, Flowers C, Roebuck E, Elston CW, Blamey RW (1990). The relationship of high risk mammographic patterns to histologic risk factors for development of cancer in the human breast. Br J Radiol.

[B27] Byrne C, Schairer C, Brinton LA, Wolfe J, Parekh N, Salane M, Carter C, Hoover R (2001). Effects of mammographic density and benign breast disease on breast cancer risk (United States). Cancer Causes Control.

[B28] Fisher ER, Palekar A, Kim WS, Redmond C (1978). The histopathology of mammographic patterns. Am J Clin Pathol.

[B29] Wellings SR, Wolfe J (1978). Correlative studies of the histological and radiographic appearance of the breast parenchyma. Radiology.

[B30] Bright R, Morrison A, Brisson J, Burstein NA, Sadowsky NS, Kopans DB, Meyer JE (1988). Relationship between mammographic and histologic features of breast tissue in women with benign biopsies. Cancer.

[B31] Urbanski S, Jensen HM, Cooke G, McFarlane D, Shannon P, Kruikov V, Boyd NF (1988). The association of histologic and radiologic indicators of breast cancer risk. Br J Cancer.

[B32] Hawes D, Downey S, Pearce CL, Bartow S, Wan P, Pike MC, Wu AH (2006). Dense breast stromal tissue greatly increased concentration of breast epithelium but no increase in its proliferative activity. Breast Cancer Res.

[B33] Stomper PC, Penetrante RB, Edge SB, Arredondo MA, Blumenson LE, Stewart CC (1996). Cellular proliferative activity of mammographic normal dense and fatty tissue determined by DNA S phase percentage. Breast Cancer Res Treat.

